# Bed bugs, *Cimex lectularius*: Undercover agents in forensic investigations

**DOI:** 10.1111/1556-4029.15638

**Published:** 2024-10-14

**Authors:** Kelly A. Meiklejohn, Coby Schal, Khalid M. Lodhi

**Affiliations:** ^1^ Department of Population Health and Pathobiology North Carolina State University, College of Veterinary Medicine Raleigh North Carolina USA; ^2^ Department of Entomology and Plant Pathology North Carolina State University, College of Agriculture and Life Sciences Raleigh North Carolina USA; ^3^ Department of Biological and Forensic Sciences Fayetteville State University Fayetteville North Carolina USA

**Keywords:** bed bugs, DNA profile, DNA testing, human blood, human male DNA, RSID blood testing, short tandem repeats (STR), Y‐STR loci

## Abstract

Insects have long played a role in forensic investigations and can be used to estimate minimum time since death, corpse translocation, and link an individual to a crime scene. Bed bugs (*Cimex lectularius*) are wingless ectoparasitic insects of potential forensic utility, given that all mobile life stages feed on vertebrate blood. Successful profiling of autosomal short tandem repeats (STRs) from human DNA isolated from bed bugs has been previously reported. This proof‐of‐concept study looked to expand this work and determine any possible limitations of using bed bugs for both rapid stain identification (RSID™) for human blood and Y‐STR profiling. To achieve this, bed bugs were fed either human male only or human pooled (female:male) blood for 30 min and subsequently collected at 12‐h intervals up to 108 h post‐blood meal (PBM). RSID™ blood testing was successful from the bed bug carcass remaining after DNA isolation, regardless of blood meal type and time of collection PBM. Complete Y‐STR profiles were generated from bed bugs <60 h PBM. As the time PBM increased, DNA quantity decreased, while the degradation index increased. Collection of bed bugs at a crime scene could provide a valuable source of human blood for Y STR profiling and be used to link an individual to a crime scene or for potential male suspect exclusion. Future studies should look to replicate the results of this proof‐of‐concept study with larger numbers of bed bugs, more diverse blood donors, and additional STR profiling kits.

**Highlights**

- Bed bugs feed on human blood and therefore they could be valuable for DNA profiling.
- Confirmatory blood testing was successful for all samples.
- Complete Y‐STR profiles were obtained from bed bugs up to 60 h post‐blood meal regarless of blood meal composition.
- Bed bugs represent a largely untapped source of human DNA for forensic casework.

## 1 INTRODUCTION

The bed bug, *Cimex lectularius* L., is one of the most widely disseminated household insect species worldwide. They are typically found in clusters within residential settings and are easily dispersed by people and can infest locations quickly [1]. Bed bugs are adapted to feed on humans to obtain blood, but they accept other hosts and were identified as an increasing threat to the U.S. poultry industry in 2011 [2, 3]. Unlike most hematophagous insects, all mobile stages of bed bugs (nymphs and adults) and both sexes feed exclusively on blood. Bed bug nymphs require a bloodmeal to grow and molt to the next stage (next instar or adult), thus feeding on humans approximately every 3–7 days [4]. Adults require a blood meal to reproduce (egg production in females and sperm production in males) [4]. If undisturbed, bed bugs will fully engorge on a single host, but they may feed on multiple human hosts in the same location if disturbed during feeding [1, 5]. Passive, human‐mediated dispersal is the most significant way bed bugs spread to a new location, transported in clothes, on shoes or in used furniture. People can also transport bed bugs when they travel by cars, trains, ships, or airplanes [1, 6].

Since 2004, the resurgence of bed bugs in the U.S. has caught the attention of forensic investigators, given that human blood in the gut of a bed bug could be utilized to identify the bed bug host(s) [6]. Further, bed bugs could be used in a criminal investigation to connect a human host to a specific location [7]. Unlike mosquitoes that can fly tens to hundreds of meters between bloodmeals, bed bugs remain in the vicinity of the host after feeding on its host [1, 8]. Thus, the human blood acquired from bed bugs could be used to link a potential victim or suspect to a crime scene.

The use of blood meals recovered from insect guts as a source of human DNA has been documented. A number of studies have successfully isolated, amplified, and genotyped human mitochondrial DNA (mtDNA) from blood‐feeding insects by using polymerase chain reaction (PCR) techniques and sequencing mtDNA hypervariable region markers. These insects include the human crab louse (*Phthirus pubis*) [9], bed bugs (*C. lectularius*), [7] and mosquitoes (*Aedes aegypti*) [10]. Additional research has also shown that human nuclear DNA (nuDNA) can be successfully isolated and complete autosomal STR profiles generated from bed bugs (*C. lectularius*) collected 72 h after ingesting a human male or a pooled (female:male) blood meal [11]. Notably, DNA did degrade over time resulting in the loss of primarily larger autosomal STR loci with longer times post‐blood meal (PBM) [11]. Nuclear DNA profiling of human blood meals from mosquitoes [12, 13, 14, 15, 16, 17, 18, 19], tropical bed bugs (*Cimex hemipterus*) [20, 21], and leeches [22] has also been tested, with the majority of these studies reporting full autosomal short tandem repeat (STR) profiles up to 24 h PBM.

While earlier research has demonstrated the ability to extract human mtDNA and nuDNA from the blood meals of insects including bed bugs, there is no research assessing whether human male DNA can be profiled from a bed bug fed on only human male or a pooled (female:male) blood meal. The use of STRs from the Y‐chromosome (herein referred to as Y‐STRs) in forensic investigations has been increasing [23, 24, 25, 26, 27, 28] and is especially important in rape, paternity, missing persons, and immigration cases. Given the Y‐chromosome is a single haplotype in most cases, only a single allele will be present for each Y‐STR locus [29, 30]. Furthermore, Y‐STRs also confer an advantage when testing samples that have female and male DNA contributors [30]. In such scenarios, Y‐STR analysis will completely disregard the female portion of the DNA, which is most common in rape cases where female DNA is the major contributor [23, 28, 30].

This study aimed to assess (a) whether bed bugs could be used for human blood identification and human Y‐STR profiling and (b) the limits for detecting a full Y‐STR profile for downstream analysis up to 108 h PBM.

## 2 MATERIALS AND METHODS

### 2.1 Human blood feeding to bed bugs

Bed bug (*C. lectularius*) colonies were maintained at North Carolina State University's Entomology laboratories (NCSU‐EL) in an incubator at 27°C, ~50% relative humidity, and 12:12‐h light:dark, as explained in Schal et al. [11]. Bed bugs were fed and collected at NCSU‐EL, as discussed in previous publications [31]. Bed bugs (*n* = 190), in two different containers, were fed human defibrinated only male human blood (*n* = 100; Bioreclamation IVT, New York, NY) or pooled (1 female:1 male, vol:vol; Bioreclamation IVT) blood (*n* = 90). Bed bugs were given 30 min to feed and then collected at 12‐h intervals PBM up to 108 h PBM. A total of 10 bed bugs were collected at each time interval and placed into individual 2.0‐mL labeled Eppendorf tubes, containing 95% ethanol and stored at −80°C. The volume of blood consumed by a fully engorged adult male bed bug in the artificial feeding system was 3.92 ± 0.21 μL (as determined gravimetrically) [32]. The frozen bed bugs were transported on dry ice to Fayetteville State University's (FSU) Forensic Science Laboratory and stored at −80°C until further testing. The Institutional Review Board (IRB) proposal for this study was approved by the FSU Human Rights in Research Committee (IRB # 2013‐P‐0039).

### 2.2 Isolation and identification of human blood from bed bugs

Prior to DNA isolation, ethanol was removed, and the bed bugs were washed three times with 500 μL of distilled water. The presence of human blood in the bed bugs was assessed using the Rapid Stain Identification of Human Blood (RSID™‐ Blood) Kit (Independent Forensics, Hillside, IL). This kit has been validated as human blood specific (i.e., no cross reactivity with animal blood) can be used with as little as 1 μL of human blood [33, 34]. After the bed bugs were thoroughly cleaned, 10 μL of Rapid Stain Identification (RSID™) (Independent Forensics) kit universal buffer was added to the tube. A clean probe was used to homogenize the bed bugs. Eppendorf tubes containing the blood and cuticle were centrifuged at 13,000 rpm for 5 min. Bed bugs tissues, including the cuticle, were packed at the bottom of the tubes post‐centrifugation. The supernatant including the blood was carefully removed from the pellet using a 20 μL pipette and transferred onto a labeled Flinders Technology Associates (FTA) micro card (GE Healthcare Bio‐Sciences Corp., Piscataway, NJ). This process (addition of 10 μL buffer, homogenization, centrifugation, and recovery of the supernatant onto the FTA card) was repeated two more times for each bed bug sample to complete the transfer of the entire volume of supernatant. The FTA card was then placed inside a sterile hood in the dark to air dry at room temperature overnight.

An additional 100 μL of RSID™ universal buffer was added to the remaining tissues and exoskeleton (pellet) in the Eppendorf tube and thoroughly vortexed, centrifuged, and incubated at room temperature for 1 h (vortexing after every 10 min). The entire 100 μL in the tube was subsequently pipetted into the RSID™ sample well cassette for Human Blood Identification. Samples were left to diffuse into the RSID™ cassette for 10 min. While the strength of the test line in immunoassays is not generally considered to be reflective of the amount of blood in a sample, a signal intensity rating between 0 and 5 was scored for each sample (0 indicating a negative reaction and 5 indicating a strong positive reaction). In addition to the intrinsic negative and positive control lines present in each test cassette to examine test efficacy, a negative and a positive control were processed alongside the test samples to ensure that the kit worked properly. Starved bed bugs not fed either on human blood or on non‐human blood served as a negative control; the RSID blood test results were negative, confirming human blood was undetected.

### 2.3 Isolation of DNA and human DNA quantitation

A minimum of two bed bugs collected from each time interval PBM that fed on either human male only blood or human pooled blood were carried forward for DNA isolation. The DNA isolation of total genomic DNA from dried blood FTA‐stained cards and quantitation of human as well as human male DNA was determined as described in Schal et al. [11]. Briefly, this involved using approximately a quarter of the dried blood FTA‐stained card as input into a phenol: chloroform: isoamyl alcohol (25:24:1; PCIA) DNA isolation. DNA was concentrated to 15 μL using Amicon® Ultra‐4 Centrifugal Filtration technique (Millipore, Billerica, MA). Human male‐specific DNA concentration was measured in duplicate using 7500 Real‐Time PCR (Thermo Fisher Scientific) in conjunction with the Quantifiler® Human DNA Duo Quantitation Kit (Applied Biosystems, Foster City, CA) [11]. To assess the level of DNA degradation, DNA isolated from selected bed bugs was further quantified in duplicate using the Quantifiler® Trio DNA Quantification Kit (Applied Biosystems) on a QuantStudio 5 Real‐Time PCR System (Applied Biosystems) as follows: a single representative from each 12‐h interval PBM collection for both only human male blood (0–108 h PBM; *n* = 10) and human pooled blood (0–96 h PBM; *n* = 9). Degradation index (DI) results provided from the Quantifiler® Trio DNA Quantification Kit were interpreted as per the manufacturer's recommendations: DI <1 typically indicates that DNA is not degraded; DI 1 to 10 typically indicates that DNA is slightly to moderately degraded; and DI >10 (or blank) typically indicates that DNA is significantly degraded [35].

### 2.4 Human male Y‐STR amplification

PCR amplification reactions for all DNA samples were prepared according to the manufacturer's recommended protocol using the AmpFLSTR™ Yfiler™ PCR Amplification Kit (Thermo Fisher Scientific; [23]). The DNA template input was 1 ng in a final 25 μL PCR. Thermal cycling was completed using a 96‐well GeneAmp® PCR System 9700 (Bio‐Rad, Hercules, CA) following the manufacturer's instructions. Negative (distilled water) and positive (007 and 9947A; Thermo Fisher Scientific) controls were amplified alongside bed bug DNA samples.

### 2.5 Human male Y‐chromosome‐specific STR profiling

All amplified samples were separated by electrophoretic capillary action by the ABI PRISM® 310 Genetic Analyzer (Thermo Fisher Scientific). ABI 310 sample preparation included 24.5 μL of Hi‐Di™ Formamide (Thermo Fisher Scientific), 0.5 μL of GeneScan™ 500 LIZ® Size Standard (Thermo Fisher Scientific), and 1 μL of PCR amplified product or AmpFLSTR™ Yfiler™ Allelic Ladder (Thermo Fisher Scientific). The reaction tubes were heated at 95°C for 3 min for a denaturation step and immediately frozen at −20°C for 3 min, and then, the amplified samples were loaded onto the genetic analyzer. The samples were separated using a 47 cm × 50 μm capillary tube (Thermo Fisher Scientific). Amplified products were electrokinetically injected for 5 s and fractionated on an ABI Prism 310 Genetic Analyzer using POP‐4® Polymer (Thermo Fisher Scientific). All samples were analyzed using GeneMapper ID® v 3.2.1 (Thermo Fisher Scientific), and an analytical threshold of 100 relative fluorescence units (RFU) was used for all dyes.

### 2.6 Statistical analyses

Raw DNA quantification data along with the output from GeneMapper ID v 3.2.1 (i.e., genotyped alleles and associated peak heights in RFU) were imported into Microsoft Excel (Microsoft, Redmond, WA, USA). Calculations included average DNA yields, allele peak heights and percentage of correct allele calls, and Pearson correlation analyses.

## 3 RESULTS AND DISCUSSION

### 3.1 Confirmation of human blood from bed bugs

To establish the presence of human blood in bed bugs, RSID™ blood testing was conducted. This kit has been validated to only give a positive result with human blood; no cross‐reactivity has been reported when testing with other human bodily fluids (e.g., saliva, semen, breast milk, amniotic fluid, vaginal fluid or urine) or blood from other species (ferret, skunk, opossum, dog, cat, cow, pig, chicken, owl, horse, goat, turtle, elk, deer, tiger, alpaca, orangutan, gorilla, spider monkey, bonobo, and baboon) [33, 34]. Human‐specific blood was identified from bed bugs fed human male only blood (intensity ranging from 4.33 to 0.5) at 0–108 h PBM (Table 1). Additionally, human‐specific blood was detected from bed bugs fed human pooled blood (intensity ranging from 4.5 to 1) at 0–96 h PBM (Table 1). The intensity of the RSID™ blood assay decreased with time PBM, with a negative correlation between intensity of the RSID™ blood assay and time PBM for both bed bug cohorts (*r* = −0.82 and −0.69; fed human male only and human pooled blood, respectively). Notably, these results also demonstrate that confirmatory RSID™ blood testing can be successful using only the trace amounts of human blood remaining on previously extracted bed bug tissues and exoskeleton.

**TABLE 1:** Summary of key metrics when assessing the utility of bed bugs fed human blood for downstream Y‐STR profiling.

Blood meal source	Collection time post‐blood meal (h)	Average RSID™ intensity^a^	Range total human male DNA yield (ng/bed bug)^b^	Degradation index^c^	Y‐STR profiling					
					Average peak height (RFU)				Average % correct allele calls	Average profile completeness
					All loci	Short loci (<165 bp)	Medium loci (166–240 bp)	Long loci (>240 bp)		
Human male only	0	3.0	2.30–2.88	1.89	909	1794	616	434	95.1	Full
	12	4.3	4.82–5.43	0.72	2020	2455	2071	1512	91.2	Full
	24	4.0	1.73–3.24	0.90	1225	1685	1062	995	76.5	Full
	36	3.7	2.72–2.73	0.64	2120	2924	1972	1525	80.0	Full
	48	1.7	1.27–1.92	0.64	1953	2378	1869	1647	82.4	Full
	60	3.5	1.10–1.42	0.80	1429	2228	1321	780	80.0	Full
	72	2.0	LOD‐0.06^d^	2.90	312	584	230	156	78.2	Full
	84	2.0	0.08–0.09^d^	0.66	1824	2604	1758	1135	61.8	Partial
	96	1.0	LOD ‐0.59^d^	2.51	90	165	55	65	41.2	Partial
	108	0.5	LOD^d^	5.24	135	258	105	53	24.1	Partial
Human pooled 1:1 (female: male)	0	2.0	0.51–0.73	2.16	951	1930	657	384	79.4	Full
	12	3.0	2.49–3.02	0.98	2127	2392	2165	1811	75.0	Full
	24	4.5	2.16–2.27	0.74	1732	2384	1722	1093	70.0	Full
	36	2.0	1.13–1.50	0.98	1207	1877	1186	566	70.0	Full
	48	2.0	0.37–0.62^d^	1.28	926	1553	789	492	67.1	Full
	60	2.0	0.18–0.24^d^	1.13	370	631	333	162	66.5	Full
	72	1.0	0.03–0.27^d^	4.29	194	392	159	44	59.4	Partial
	84	1.0	0.01–0.13^d^	3.17	62	141	40	12	41.2	Partial
	96	1.0	LOD^d^	3.80	123	243	90	51	30.6	Partial

*Note*: Averages based on two bed bugs.

Abbreviations: bp, base pair; h, hour; LOD, below limit for detection (23 pg/μL); RFU, relative fluorescence units; RSID™, Rapid Stain Identification for Blood; STR, short tandem repeat.

^a^ Denotes signal intensity rating between 0 and 5 (where 0 indicates a negative [not detected] and 5 indicates a strong positive reaction); average among 10 replicates.

^b^ Denotes based on Quantifiler Human DNA Duo Quantitation Kit (Applied Biosystems) 130 bp male target.

^c^ Denotes based on ratio of long to short autosomal targets determined using Quantifiler Trio DNA Quantification Kit (Applied Biosystems) based on duplicate amplifications from a single representative bed bug DNA extract.

^d^ Denotes that less than the optimal 1 ng of DNA was used as input for Y‐STR profiling.

### 3.2 DNA quantitation from bed bugs

The total human male‐specific DNA yield from bed bugs fed solely on human male blood ranged from below the limit of detection for the assay (i.e., 23 pg/μL) up to 5.43 ng (Table 1). The total human male‐specific DNA recovered from bed bugs fed on pooled (female: male) blood was lower than for bed bugs fed male only blood; ranged from below the limit of detection for the assay to 3.02 ng (Table 1). For both bed bug cohorts, the highest human male DNA was recovered from bed bugs collected 12 h PBM. This is consistent with our preliminary observations that cell lysis is initiated shortly after the blood is ingested (Wiles and Schal, personal observations), which would facilitate DNA isolation from leukocytes. Given the suggested DNA quantity input for AmpFLSTR™ Yfiler™ PCR Amplification Kit is 1 ng (with a maximum DNA input volume of 10 μL), only bed bugs collected up to 60 h PBM for male only fed and 36 h PBM pooled fed bed bugs had the optimal DNA input for amplification (Table 1). Strategies commonly employed to further concentrate DNA (e.g., drying down via SpeedVac or bead purification) still would not permit optimal DNA input for all samples, given the overall low yields per bed bug (Table 1). However, higher DNA yields might be obtained when using a more efficient DNA isolation method, such as a bead beating grinder and lysis instrument. Overall, our DNA yields are consistent with preliminary observations that lysis of erythrocytes, and likely leukocyte lysis as well, is almost complete at 72 h PBM and hemoglobin concentrations in the bed bug plummet between 24 and 72 h PBM, suggesting extensive degradation of DNA as well (Wiles and Schal, preliminary observations).

### 3.3 Human male Y‐chromosome‐specific STR profiling

Profiling of Y‐STRs from DNA isolated from bed bugs collected up to 108 h PBM was successful, establishing that adequate intact human male‐specific DNA can be isolated, amplified, and profiled from a single bed bug. Human male‐specific Y‐STR profiles from each bed bug sample were evaluated against the human male reference blood DNA Y‐STR profile to assess whether complete and/or partial match or no profile was recovered at 12‐h intervals from 0 to 108 h PBM. Complete human Y‐STR profiles were obtained from bed bugs that were fed on either human male only blood or pooled blood until 72 h and 60 h PBM, respectively (Table 1); only partial profiles were recovered for time points beyond. Given there is a continuous process of cell lysis followed by digestion of cell contents PBM, it is possible that at time points beyond 60 h PBM, these processes greatly diminished available leukocytes for DNA isolation. Notably, full profiles were recovered when up to 50‐fold less DNA than suggested for amplification using AmpFLSTR™ Yfiler™ PCR Amplification was used. For example, bed bugs fed male only human blood and collected 72 h PBM only had 0.02 ng total DNA as input for amplification, but a full profile was obtained (Table 1). When examining whether allele calls were correct (i.e., DNA profile(s) generated from a bed bug matches with the DNA profile of the reference human blood DNA) (Supplemental Figure S1), we noted that (a) >80% of calls were correct for bed bugs fed single source human male only blood and collected up to 72 h PBM, (b) >70% of calls were correct for bed bugs fed human pooled blood and collected up to 36 h PBM, and (c) there were no incorrect allele calls. While a moderate positive correlation was observed between RSID blood testing results and the percentage of correct allele calls for bed bugs fed male only and pooled blood (*r* = 0.78 and 0.60, respectively), additional experiments are needed with larger samples to confirm this association.

The average PBM human DNA concentration decreased overtime (0‐108 h from bed bugs fed on human male and 0‐96 h from bed bugs fed on human pooled blood) except for at 12 h (Table 1). In order to relate the quantity of recovered human DNA to Y‐STR markers peak height, we compared over time four exemplar Y‐STR markers representing short (<165 bp; DYS456), medium (166–240 bp; DYS391 and DYS635), and large length loci (>240 bp; DYS392) (Supplemental Figure S2); overall, a decrease in peak height was observed with both time PBM and locus length. A side‐by‐side comparison of peak heights for all Y‐STR loci typed across all samples and time points is shown in Supplemental Figure S3. The average RFU for all loci, short length loci (<165 bp; DYS456, DYS393, Y_GATA_H4, DYS458, DYS389I), medium length loci (166–240 bp; DYS391, DYS437, DYS19, DYS439, DYS390, DYS438, DYS635), and large length loci (>240 bp; DYS385a, DYS385b, DYS389II, DYS448, DYS392) across both bed bug cohorts and time PBM are shown in Table 1. When an analytical threshold of 100 RFU was applied, both medium and large length loci fell below the threshold for bed bugs collected after 96 or 84 h PBM for male only and pooled, respectively. Notably, when a lower analytical threshold was applied (e.g., 50 RFU), more complete Y‐STR profiles were possible for longer times PBM (Table 1). These data indicate that while the optimal time PBM for the collection of bed bugs to yield a complete Y‐STR profile is <60 h, a partial profile mostly consisting of shorter length loci can be generated 4–5 days PBM which could be valuable for male suspect exclusion.

### 3.4 Degradation index

The average DI from duplicate reactions of a single bed bug from each time point PBM is shown in Table 1. A moderate positive correlation between time PBM and DI was observed for bed bugs fed male only and pooled blood (*r* = 0.59 and 0.68, respectively), indicating that DNA degradation increases with time PBM. For both bed bug cohorts, negative correlations were obtained between DI and average peak heights based on loci length (all, short, medium, and large), demonstrating that as DI increases, average peak heights decrease. A strong negative correlation was observed with time PBM and the percentage of correct allele calls (*r* = −0.73 and − 0.70; fed human male only blood and human pooled blood, respectively). It should be noted that correlation analyses using DI values were completed using data generated from only a single bed bug at each time interval; experiments should be repeated with more bed bugs from each blood meal type and time interval to confirm whether these trends still hold. Additionally, when determining whether as sample is degraded, consideration should be given to the pattern of the profile (e.g., consistent peak heights across short and long loci or a “ski slope” pattern) and the relationship between DNA input quantity and allele peak height.

## 4 CONCLUSIONS AND LIMITATIONS

This proof‐of‐concept study aimed to assess whether bed bugs represent a suitable source of human blood for identification and Y‐STR profiling, along with the subsequent limits for detecting a full Y‐STR profile for downstream analysis. The key takeaways are as follows: (a) RSID™ blood testing was successful from the bed bug tissues (carcass including cuticle) remaining after DNA isolation, regardless of blood meal type (human male only or pooled female: male blood) and time of collection, (b) quantifiable male human DNA was isolated from bed bugs that were fed on either human male only blood or pooled blood 84 h PBM, (c) complete Y‐STR profiles were generated from bed bugs that were fed on either human male only blood or pooled blood until 72 h and 60 h PBM, respectively, and (d) as the time PBM increased, allele peak heights and RSID™ blood intensity decreased, while the DNA DI increased. Notably, bed bugs in this study were collected into 95% ethanol and immediately stored at −80°C. To simulate crime scene conditions, future work should examine the impact of varied storage conditions (e.g., room temperature, paper bag storage as opposed to ethanol) on the recovery of both autosomal and Y‐STR profiles. A major caveat is that our bed bugs were incubated at 27°C. Lower average temperatures in homes would slow down blood digestion and DNA degradation and extend the PBM period of quantifiable human DNA. Future studies should look to replicate the results of this proof‐of‐concept study with larger numbers of bed bugs, more diverse blood donors, and additional STR profiling kits. Moreover, in this study we used an artificial feeding system, defibrinated human blood, and fully fed (engorged) bed bugs. Although blood meal sizes are similar on anti‐coagulant‐treated and whole blood [36], future studies should examine bed bugs that fed directly on humans, including bed bugs that ingested partial blood meals. These results highlight the potential of a largely overlooked source of human blood as well as human genomic DNA at a crime scene, and investigators should consider collecting bed bugs if encountered.

## FUNDING INFORMATION

Funding received from the National Science Foundation, Directorate for STEM Education, Division of Undergraduate Education, #0714826 and #1719511.

## CONFLICT OF INTEREST STATEMENT

The authors have no conflicts of interest to report.

## Supporting information


Figure S1.

Figure S2.

Figure S3.

## References

[1] Reinhardt K , Siva‐Jothy MT . Biology of the bed bugs (Cimicidae). Annu Rev Entomol. 2007;52:351–374. 10.1146/annurev.ento.52.040306.133913 16968204

[2] Cater J , Magee D , Hubbard SA , Edwards KT , Goddard J . Severe infestation of bed bugs in a poultry breeder house. J Am Vet Med Assoc. 2011;239(7):919–922. 10.2460/javma.239.7.919 21961628

[3] González‐Morales MA , Thomson AE , Petritz OA , Crespo R , Haija A , Santangelo RG , et al. Systemic veterinary drugs for control of the common bed bug, *Cimex lectularius*, in poultry farms. Parasit Vectors. 2002;15(1):431. 10.1186/s13071-022-05555-6 PMC9670615 36397113

[4] Miller DM , Polanco A , Rogers J . Bed bug biology and behavior. Virginia Cooperative Extension. 2019 https://vtechworks.lib.vt.edu/server/api/core/bitstreams/c51e4e67‐c19d‐44cc‐a90b‐1b9c51e998cc/content Accessed 29 Aug 2024.

[5] Koganemaru R , Miller DM . The bed bug problem: past, present, and future control methods. Pestic Biochem Physiol. 2013;106(3):177–189. 10.1016/j.pestbp.2013.05.005

[6] Alalawi AH . Bed bugs epidemic in the United States. Entomol Ornithol Herpetol. 2015;04(1):1000143. 10.4172/2161-0983.1000143

[7] Szalanski A , Austin J , McKern J , McCoy T , Miller D . Time course analysis of bed bug, *Cimex lectularius* L., (Hemiptera:Cimicidae) blood meals with the use of polymerase chain reaction. J Agric Urban Entomol. 2006;23(4):237.

[8] Greenberg JA , DiMenna MA , Hanelt B , Hofkin BV . Analysis of post‐blood meal flight distances in mosquitoes utilizing zoo animal blood meals. J Vector Ecol. 2012;37(1):83–89. 10.1111/j.1948-7134.2012.00203.x 22548540 PMC3342775

[9] Lord WD , DiZinno JA , Wilson MR , Budowle B , Taplin D , Meinking TL . Isolation, amplification, and sequencing of human mitochondrial DNA obtained from human crab louse, *Pthirus pubis* (L.), blood meals. J Forensic Sci. 1998;43(5):1097–1100. 10.1520/jfs14367j 9729835

[10] Vieira BRC , Carvalho EF , Silva DA . Analysis of human DNA present in the digestive tract of *Aedes aegypti* mosquitoes for possible forensic application. Forensic Sci Int Genet Suppl Ser. 2017;6:e324–e326. 10.1016/j.fsigss.2017.09.124

[11] Schal C , Czado N , Gamble R , Barrett A , Weathers K , Lodhi KM . Isolation, identification, and time course of human DNA typing from bed bugs, *Cimex lectularius* . Forensic Sci Int. 2018;293:1–6. 10.1016/j.forsciint.2018.10.008 30390476

[12] Kreike J , Kampfer S . Isolation and characterization of human DNA from mosquitoes (Culicidae). Int J Leg Med. 1999;112(6):380–382. 10.1007/s004140050018 10550599

[13] Rabêlo KCN , Albuquerque CMR , Tavares VB , Santos SM , Souza CA , Oliveira TC , et al. Trace samples of human blood in mosquitoes as a forensic investigation tool. Genet Mol Res. 2015;14(4):14847–14856. 10.4238/2015.November.18.50 26600546

[14] Ahmed AM , Alotaibi AM , Al‐Qahtani WS , Tripet F , Amer SA . Forensic DNA analysis of mixed mosquito blood meals: STR profiling for human identification. Insects. 2023;14(5):467. 10.3390/insects14050467 37233095 PMC10231104

[15] Chow‐Shaffer E , Sina B , Hawley WA , De Benedictis J , Scott TW . Laboratory and field evaluation of polymerase chain reaction‐based forensic DNA profiling for use in identification of human blood meal sources of *Aedes aegypti* (Diptera: Culicidae). J Med Entomol. 2020;37(4):492–502. 10.1603/0022-2585-37.4.492 10916289

[16] Curic G , Hercog R , Vrselja Z , Wagner J . Identification of person and quantification of human DNA recovered from mosquitoes (Culicidae). Forensic Sci Int Genet. 2014;8(1):109–112. 10.1016/j.fsigen.2013.07.011 24315597

[17] Gray SL , Tiedge TM , Butkus JM , Earp TJ , Lindner SE , Roy R . Determination of human identity from *Anopheles stephensi* mosquito blood meals using direct amplification and massively parallel sequencing. Forensic Sci Int Genet. 2020;48:102347. 10.1016/j.fsigen.2020.102347 32683318 PMC7487007

[18] Ibrahim AMA , Alrakan LA , Alaifan SA , Al‐Mekhlafi FA , Kassab AC . Temporal analysis of 16 STR loci in human blood drawn from two culicid mosquitoes cultured at different temperatures. Entomol Res. 2015;45(5):268–274. 10.1111/1748-5967.12123

[19] Rabêlo KCN , Albuquerque CMR , Tavares VB , Santos SM , Souza CA , Oliveira TC , et al. Detecting multiple DNA human profile from a mosquito blood meal. Genet Mol Res. 2016;15(3):467. 10.4238/gmr.15037547 27706606

[20] Lim L , Ab Majid AH . Isolation and characterization of human DNA recovered from *Cimex hemipterus* (F.) (Hemiptera:Cimicidae). Forensic Sci Med Pat. 2020;16(4):664–670. 10.1007/s12024-020-00318-0 33159287

[21] Lim L , Ab Majid AH . Human profiling from STR and SNP analysis of tropical bed bug, *Cimex hemipterus*, for forensic science. Sci Rep. 2023;13(1):1506. 10.1038/s41598-023-28774-y 36707655 PMC9883228

[22] Knapp E , Cappas V , Roy R . Generating human STR DNA profiles from blood ingested by leeches. Can Soc Forensic Sci. 2024;57(1):20–35. 10.1080/00085030.2023.2177392

[23] Mulero JJ , Chang CW , Calandro LM , Green RL , Li Y , Johnson CL , et al. Development and validation of the AmpFℓSTR® Yfiler™ PCR amplification kit: a male specific, single amplification 17 Y‐STR multiplex system. J Forensic Sci. 2006;51(1):64–75. 10.1111/j.1556-4029.2005.00016.x 16423225

[24] Coble MD , Hill CR , Butler JM . Haplotype data for 23 Y‐chromosome markers in four U.S. population groups. Forensic Sci Int Genet. 2013;7(3):e66–e68. 10.1016/j.fsigen.2013.03.006 23578807

[25] Purps J , Siegert S , Willuweit S , Nagy M , Alves C , Salazar R , et al. A global analysis of Y‐chromosomal haplotype diversity for 23 STR loci. Forensic Sci Int Genet. 2014;12:12–23. 10.1016/j.fsigen.2014.04.008 24854874 PMC4127773

[26] Roewer L , Andersen MM , Ballantyne J , Butler JM , Caliebe A , Corach D , et al. DNA commission of the International Society of Forensic Genetics (ISFG): recommendations on the interpretation of Y‐STR results in forensic analysis. Forensic Sci Int Genet. 2020;48:102308. 10.1016/j.fsigen.2020.102308 32622324

[27] Steffen CR , Huszar TI , Borsuk LA , Vallone PM , Gettings KB . A multi‐dimensional evaluation of the ‘NIST 1032’ sample set across four forensic Y‐STR multiplexes. Forensic Sci Int Genet. 2022;57:102655. 10.1016/j.fsigen.2021.102655 35007854 PMC9901497

[28] Thompson JM , Ewing MM , Frank WE , Pogemiller JJ , Nolde CA , Koehler DJ , et al. Developmental validation of the PowerPlex® Y23 system: a single multiplex Y‐STR analysis system for casework and database samples. Forensic Sci Int Genet. 2013;7(2):240–250. 10.1016/j.fsigen.2012.10.013 23337322

[29] Kayser M . Forensic use of Y‐chromosome DNA: a general overview. Hum Genet. 2017;136(5):621–635. 10.1007/s00439-017-1776-9 28315050 PMC5418305

[30] Kayser M , Ballantyne KN . Y chromosome in forensic science. In: Primorac D , Schanfield M , editors. Forensic DNA applications. 2nd ed. Boca Raton, FL: CRC Press; 2023. p. 87–112.

[31] Romero A , Schal C . Blood constituents as phagostimulants for the bed bug *Cimex lectularius* L. J Exp Biol. 2014;217(4):552–557. 10.1242/jeb.096727 24198260

[32] Sierras A , Schal C . Comparison of ingestion and topical application of insecticides against the common bed bug, *Cimex lectularius* (Hemiptera: Cimicidae). Pest Manag Sci. 2017;73(3):521–527. 10.1002/ps.4464 27766740 PMC5288133

[33] Independent Forensics . Rapid stain identification of human blood (RSID‐blood). 2016 Available from: https://www.ifi‐test.com/documents/BLOOD‐Dual‐Buffer‐with‐SpinEZE‐042016.pdf. [Accessed 29 Aug 2024].

[34] Schweers BA , Old J , Boonlayangoor PW , Reich KA . Developmental validation of a novel lateral flow strip test for rapid identification of human blood (rapid stain identification™‐blood). Forensic Sci Int Genet. 2008;2(3):243–247. 10.1016/j.fsigen.2007.12.006 19083828

[35] Thermo Fisher Scientific . Quantifiler™ HP and Trio DNA Quantification Kits user guide. 2018 Available from: https://assets.thermofisher.com/TFS‐Assets/LSG/manuals/4485354.pdf. [Accessed 29 Aug 2024]

[36] Sasínková M , Křemenová J , Chajma P , Bartonička T , Massino C , Otti O , et al. Changes in body size and fertility due to artificial and natural feeding of laboratory common bed bugs (Heteroptera: Cimicidae). J Med Entomol. 2024;61(1):34–45. 10.1093/jme/tjad083 37889860

