# Supplementary figures and images for: Bed bugs, Cimex lectularius: Undercover agents in forensic investigations
Source: J Forensic Sci. 2024 Oct 14;70(1):264–70. doi: 10.1111/1556-4029.15638 (PMC11693522; doi:10.1111/1556-4029.15638)

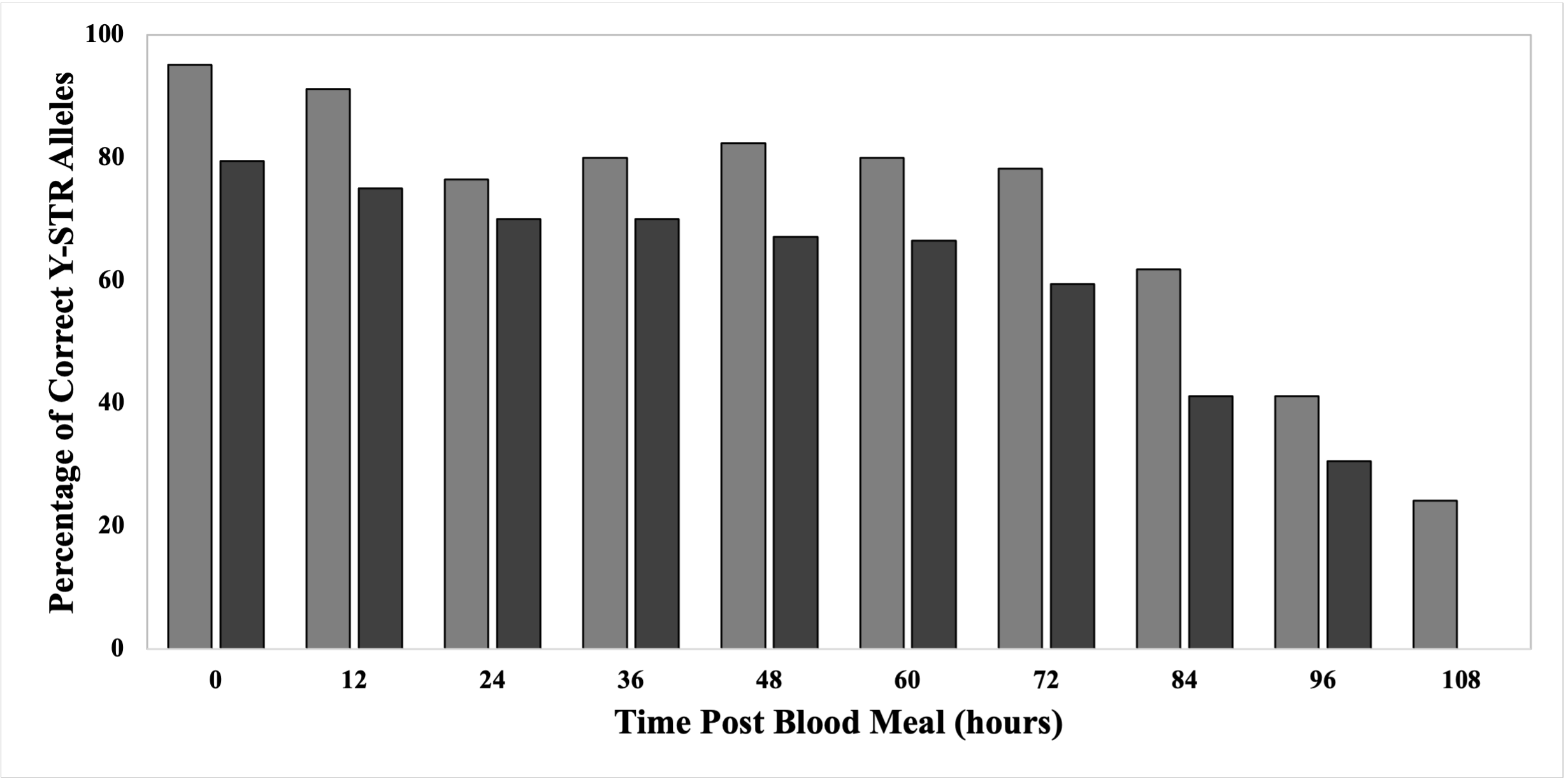

Supplement: Supplementary file 1 — Figure S1. [file JFO-70-264-s001.tiff]

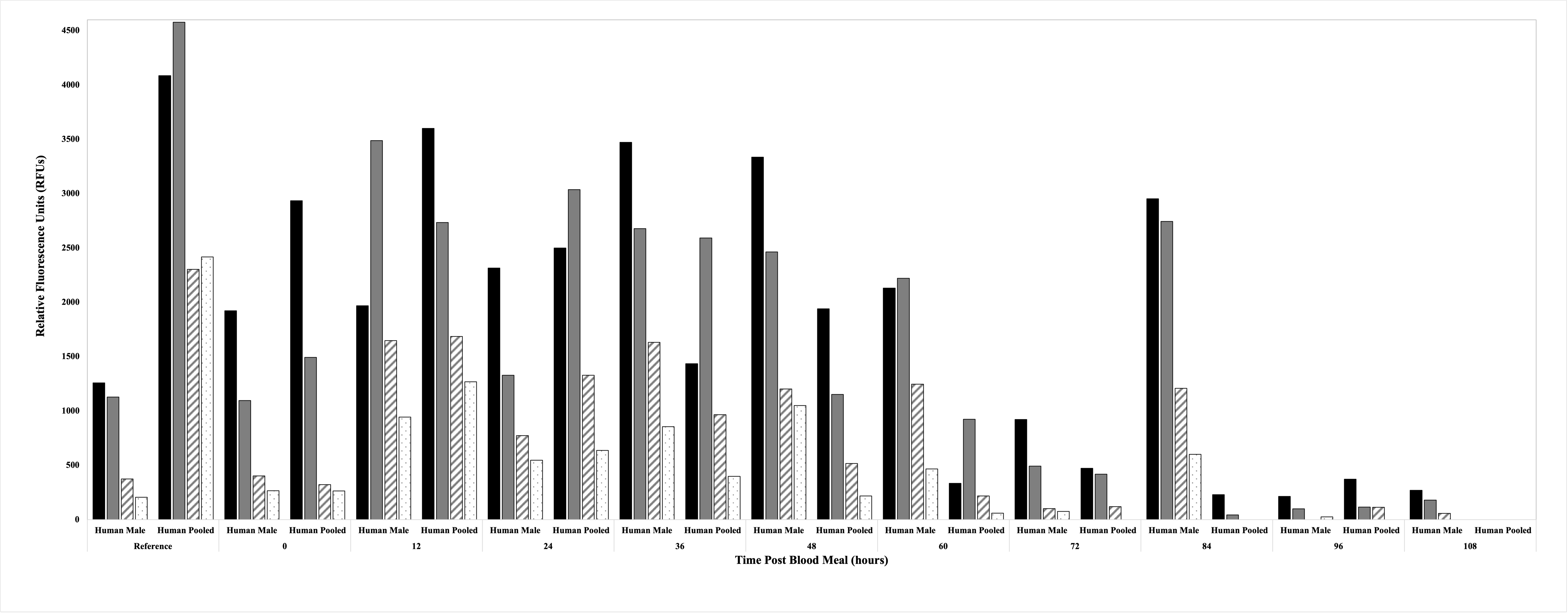

Supplement: Supplementary file 2 — Figure S2. [file JFO-70-264-s003.tiff]

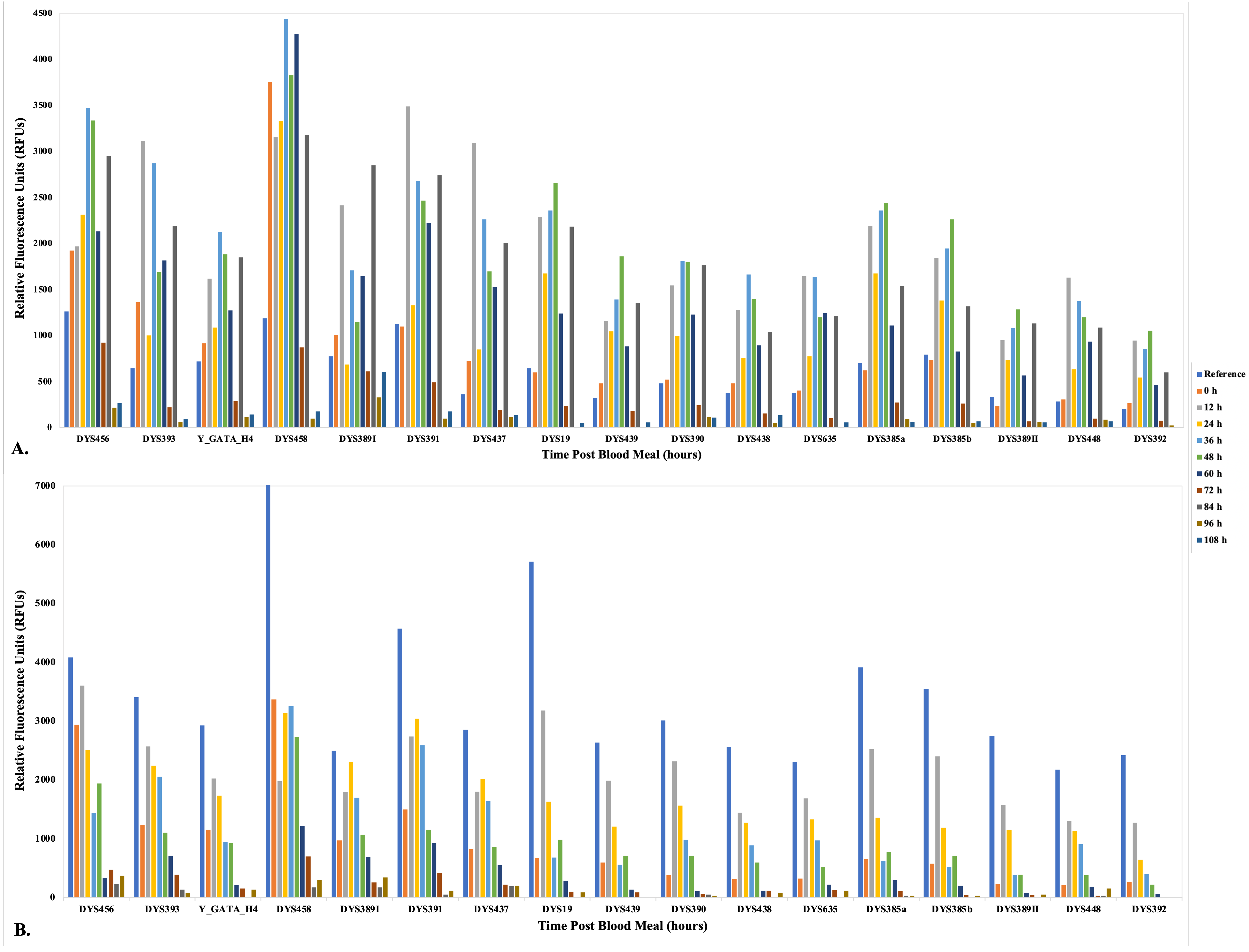

Supplement: Supplementary file 3 — Figure S3. [file JFO-70-264-s002.tiff]
